# 

**Published:** 1891-06

**Authors:** 


### SECOND CONGRESS FOR THE STUDY OF TUBERCULOSIS.

The Second Congress for the study of tuberculosis, will meet in Paris, from the 27th of July to the 2nd of August, 1891, under the Presidency of Professor Villemin. The following questions will be discussed :—

1. The identity of tuberculosis in man and tuberculosis in the bovine, gallinaceous, and other animals.

2. The bacterien and morbid associations of tuberculosis.

3. The quarantine (Hospitalisation) of tuberculosis.

4. Prophylaxy of human and animal tuberculosis.

5. Agents capable of destroying the bacillus of Koch, not harmful to the organism, from the point of view of prophylaxy and therapeutics of human and animal tuberculosis.

Subscriptions to the Congress, 20 francs, should be sent to M. G. Masson, Treasurer, 120 Boulevard St., Germain.

Communications should be sent to Dr. L. H. Petit, General Secretary, 11 Rue Monge.

### BOOKS AND PAMPHLETS RECEIVED.

The 13th Annual Report of the State Board of Health, of the State of Connecticut, for the year ending November 30, 1890, with the registration report for 1889, relating to Births, Marriages, Deaths and Divorces. Printed by the order of the Legislature, New Haven, Conn., 327 pages. In addition to other matter of general interest, is an article of 27 pages on Adulterated Food in Connecticut.

Annual Report of the Board of Regents of the Smithsonian Institution to July 1889, Washington, Government printing office 1890.

Transactions of the 22nd and 23d Meeting of the Kansas Academy of Science 1889, vol. xii, part 1. Topeka, 1890, 189 pages, contains a study on the classification of the sensations of smell, By W. S. Franklin.

